# Human Primary Dermal Fibroblasts Interacting with 3-Dimensional Matrices for Surgical Application Show Specific Growth and Gene Expression Programs

**DOI:** 10.3390/ijms22020526

**Published:** 2021-01-07

**Authors:** Sarah Grossi, Annalisa Grimaldi, Terenzio Congiu, Arianna Parnigoni, Giampiero Campanelli, Paola Campomenosi

**Affiliations:** 1 Department of Biotechnology and Life Sciences, University of Insubria, DBSV, Via J.H. Dunant 3, 21100 Varese, Italy; s.grossi1@uninsubria.it (S.G.); annalisa.grimaldi@uninsubria.it (A.G.); a.parnigoni@uninsubria.it (A.P.); 2 Department of Surgical Sciences, University of Cagliari, 09100 Cagliari, Italy; terenzio.congiu@unica.it; 3 Milano Hernia Center, Department of Surgical Science, Istituto Clinico Sant’Ambrogio, Via Luigi Giuseppe Faravelli 16, 20149 Milan, Italy; giampiero.campanelli@uninsubria.it; 4 Department of Medicine and Surgery, University of Insubria, DMC, Via Guicciardini 9, 21100 Varese, Italy

**Keywords:** 3D-biomaterials, human primary fibroblasts, gene expression, cell growth, surgery

## Abstract

Several types of 3-dimensional (3D) biological matrices are employed for clinical and surgical applications, but few indications are available to guide surgeons in the choice among these materials. Here we compare the in vitro growth of human primary fibroblasts on different biological matrices commonly used for clinical and surgical applications and the activation of specific molecular pathways over 30 days of growth. Morphological analyses by Scanning Electron Microscopy and proliferation curves showed that fibroblasts have different ability to attach and proliferate on the different biological matrices. They activated similar gene expression programs, reducing the expression of collagen genes and myofibroblast differentiation markers compared to fibroblasts grown in 2D. However, differences among 3D matrices were observed in the expression of specific metalloproteinases and interleukin-6. Indeed, cell proliferation and expression of matrix degrading enzymes occur in the initial steps of interaction between fibroblast and the investigated meshes, whereas collagen and interleukin-6 expression appear to start later. The data reported here highlight features of fibroblasts grown on different 3D biological matrices and warrant further studies to understand how these findings may be used to help the clinicians choose the correct material for specific applications.

## 1. Introduction

Prosthetic abdominal wall surgical repair is a common procedure for the treatment of several types of hernias or lesions of the muscular inner body walls. About one million prostheses for abdominal wall repair per year are used worldwide, and, since the first description of the use of a synthetic mesh for this surgery, plenty of new materials have been introduced as repairing options, leading to a considerable reduction in recurrence rates [1]. Moreover, prosthetic materials are increasingly used for other applications, such as mammary plastic surgery [2].

These materials can be synthetic or of biological derivation. Synthetic meshes are generally made of polypropylene, polyethylene-terephthalate, polytetrafluoroethylene, polyester, or polyvinylidene-fluoride, and their features depend on the material weight and size of pores [3,4]. They confer strength and stability to the abdominal wall. However, synthetic meshes can give rise to fibrotic responses and adherence. Moreover, their use in surgery at risk for infection (classified by the Centers for Disease Control and Prevention as “clean-contaminated”) is debated [5]. Biological 3D matrices are derived from the extracellular matrix (ECM) of a variety of tissues and species. They undergo sterilization, albeit different procedures can be used to this aim, and can be further processed by crosslinking. All these processes can lead to alterations of the matrix and, therefore, once implanted in the patient, affect the pathways inducing foreign body response (FBR) [6]. Other characteristics considered specifically in the instance of biological meshes include resistance to microbial infection, the ability to provide a barrier to visceral adhesion formation, and the capacity to respond in a fashion similar to native tissue [7,8].

A proper body-mesh response is fundamental for successful abdominal wall repair, but the mechanisms underlying the interactions between the 3D matrices and patient cells and tissues are still not clear and need to be further investigated [3,9,10]. A hypothesis is that operational outcome during the use of biological prostheses depends on the growth of patient cells. The balance between ECM synthesis and degradation may ultimately contribute to the success of hernia repair.

However, the number of studies dealing with the biology of these materials and their interaction with host tissues is not as wide as one could expect. Moreover, scarce information is available on the application of one or the other biological matrices in relation to specific clinical indications [11,12].

To identify possible differences among specific 3D matrices commonly used in abdominal wall repair that may guide the choice of the best one suited for specific applications, thus improving clinical outcomes, in this study, we investigated fibroblast-matrix interactions by morphological analysis and by monitoring cell proliferation. We also aimed to identify the molecular pathways activated by fibroblasts during the first phases of their interaction with the different types of 3D biological matrices. In particular, we investigated the expression of genes involved in the degradation or biosynthesis of collagen, as well as some cytokines and markers of differentiation into myofibroblasts. Human primary fibroblasts have been used for this study because of their central role in foreign body reaction and wound healing process [3,10].

## 2. Results

### 2.1. Growth of Fibroblasts on the Different 3D Matrices

#### 2.1.1. Morphological Examination by SEM

**Figure 1:**
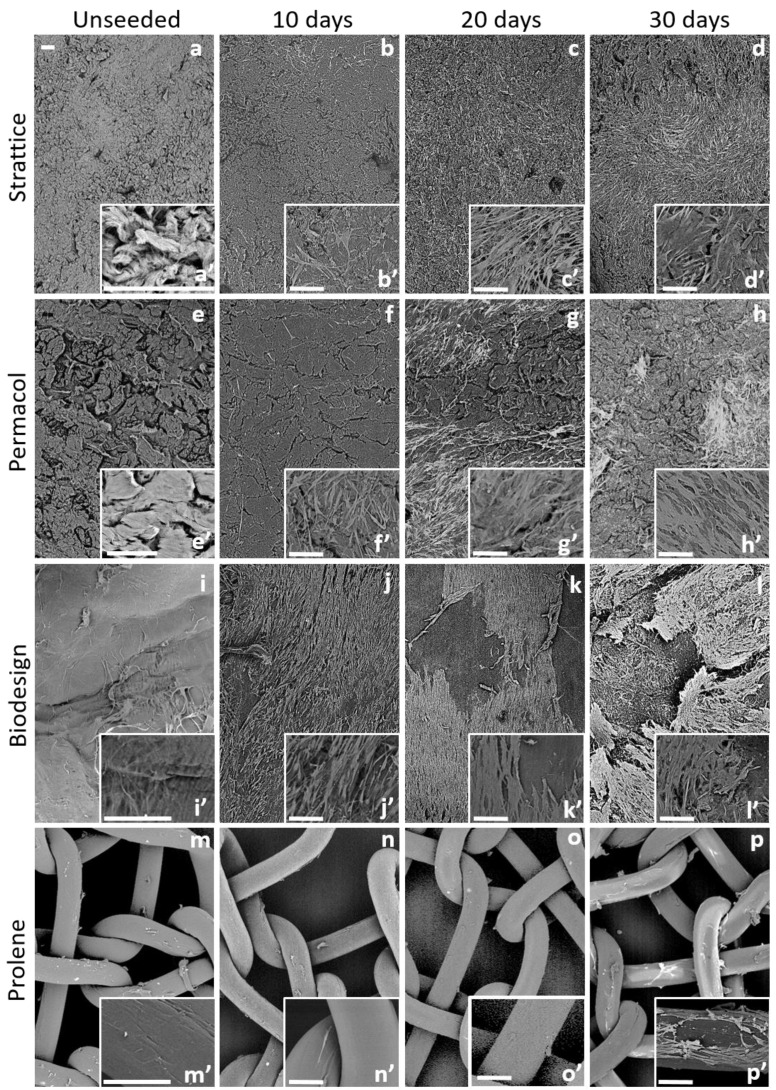
Scanning Electron Microscopy (SEM) images of human dermal fibroblasts cultured on tested matrices for 10, 20, or 30 days and corresponding unseeded controls, as indicated above the panels. Inserts represent images at higher magnification. Strattice (**a**–**d**,**a’**–**d’**), Permacol (**e**–**h**,**e’**–**h’**), Biodesign (**i**–**l**,**i’**–**l’**), Prolene (**m**–**p**,**m’**–**p’**). Bars indicate 100 μm.

The morphology of fibroblasts grown on different types of 3D matrices was analyzed by scanning electron microscopy (SEM) 10, 20, and 30 days after their seeding. Three independent experiments were carried out, and exemplificative pictures are shown in Figure 1.

The surface of the Strattice biomaterial appeared non-homogeneous: irregular areas alternated with smoother ones, suggesting a multidirectional organization of collagen fibers in the scaffold (Figure 1a’). Ten days after seeding, few cells were visible on the Strattice mesh surface, and they were not uniformly distributed (Figure 1b’). The number of cells progressively increased at 20 and 30 days; however, a non-homogeneous distribution was also observed at these time points (Figure 1c,d). Cells appeared to be healthy and seemed to firmly adhere to the matrix, as shown in Figure 1b’–d’.

The surface of the Permacol scaffold appeared similar to Strattice, alternating rough and smooth areas (Figure 1e’). The number of cells at 10 days was higher on Permacol than on Strattice and progressively increased at 20 and 30 days. However, a non-homogeneous distribution was also observed, with groups of healthy cells scattered on the scaffold surface (Figure 1f–h,f’–h’).

The surface of the Biodesign scaffold appeared smoother than Strattice and Permacol (Figure 1i’). This matrix presented the greatest number of cells among all matrices at all analyzed times. After 10 days from seeding, cells covered the surface of the biomaterial homogeneously (Figure 1j). At 20 days, cells appeared in close contact with each other, forming a monolayer (Figure 1k). However, they did not seem to firmly adhere to the scaffold, since at 30 days, the monolayer seemed to bend out and to detach from the matrix (Figure 1l). Details at higher magnification are provided in Figure 1j’–l’.

Finally, we observed very few cells adhering to the Prolene small pore synthetic matrix at all times. Cells did not seem to easily attach and grow on this material. At 30 days, only a few cells were present on the scaffold (Figure 1n–p,n’–p’).

High magnification images are also shown in Supplementary Figure S1.

#### 2.1.2. Cell Growth Curves

**Figure 2:**
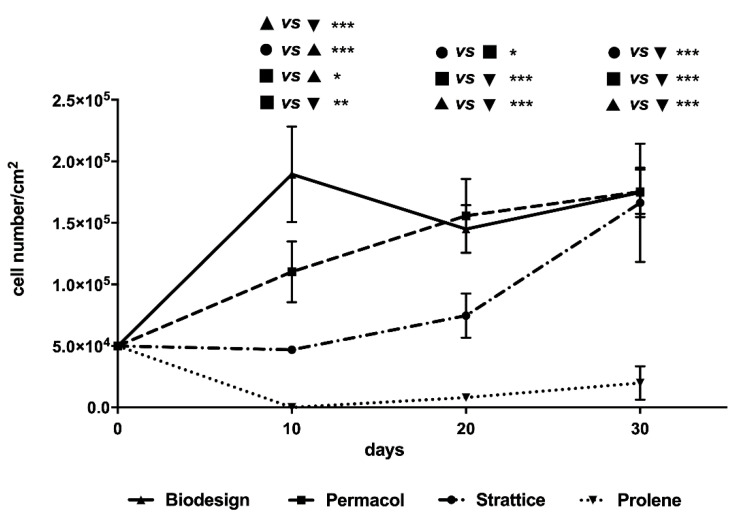
Growth of human dermal fibroblasts on tested matrices at 10, 20, and 30 days. Biodesign: line with upwards pointing triangles; Permacol: dashed line with squares; Strattice: dash-dot line with circles; Prolene: dotted line with downwards pointing triangles. Average number of cells (± SE) obtained at each time point with two methods (Cell Titer Glo Promega kit and manual count after detachment with an enzymatic cocktail) in three independent experiments were analyzed with analysis of variance (ANOVA) using a *post-hoc* Bonferroni test. Asterisks indicate significant differences (* *p* < 0.05; ** *p* < 0.01; *** *p* < 0.001).

The growth of cells on the different 3D matrices was monitored at the three chosen time points by two different methods, as detailed in Materials and Methods. Data presented are the averages of three independent experiments taking into account the values obtained by the two methods used for cell counting for each matrix at each time point (Figure 2).

At 10 days from seeding, the Biodesign mesh showed the highest number of cells among all tested scaffolds. At 20 days, a slight, temporary decrease in cell number compared to 10 days was observed on this matrix, whereas at 30 days, the number of cells was similar to that observed at 10 days (line with upwards pointing triangles). On Permacol scaffolds, cells increased in numbers from seeding to 30 days (dashed line with squares). On Strattice, the number of cells at 10 days was the same seeded at the beginning of the experiment; then it increased until it reached the number present on the other biological matrices at 30 days (dash-dot line with circles).

Indeed, whereas at 10 days, the number of cells was higher on Biodesign, followed by Permacol and Strattice, at 30 days, the number of cells grown on biological scaffolds was comparable. Very few cells were recovered in the Prolene samples, in keeping with morphological observation of a lower number of cells attaching to this synthetic mesh. However, the cell number showed a modest increase over time (dotted line with downwards pointing triangles).

### 2.2. Gene and Protein Expression

Although in our study we included for comparison a synthetic mesh together with biological matrices, gene and protein expression analyses from fibroblasts grown on Prolene were impaired by the small number of cells recovered from this type of mesh.

For all 3D matrices, no difference was observed in the results obtained with fibroblasts from the two donors; therefore, the results were pooled together.

#### 2.2.1. Expression of Collagen Genes

**Figure 3:**
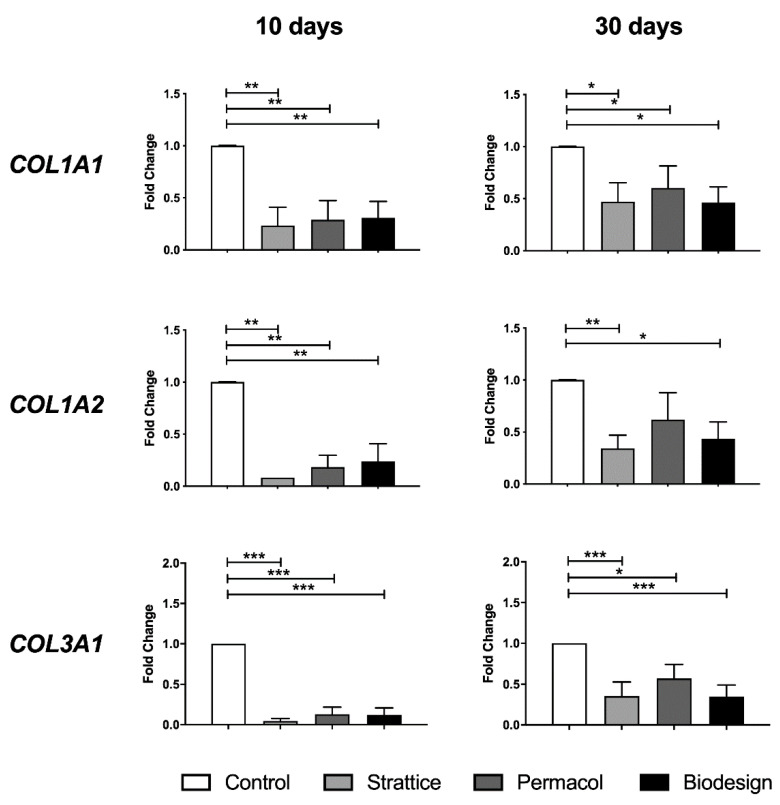
Changes in the levels of collagen transcripts following fibroblast growth on the different types of matrices for 10 or 30 days compared to the control condition—expression from fibroblasts grown on plastics (control), Strattice, Permacol, and Biodesign. Values are the means of four independent experiments ± standard error (SE). The results were analyzed with ANOVA. Asterisks indicate significant differences (* *p* < 0.05; ** *p* < 0.01; *** *p* < 0.001).

Fibroblasts grown on the different types of mesh may activate either new matrix deposition or degradation of the scaffold as a first transcriptional program. Therefore, we examined collagen I and collagen III production, the types of collagen produced by primary dermal fibroblasts used in this study. At 10 days, fibroblasts showed a significant decrease in all collagen transcripts when grown on all meshes compared to those grown on plastics (*p* < 0.005, ANOVA using a *post-hoc* Duncan’s test) (Figure 3, left panel). At 30 days, collagen transcript levels in cells grown on meshes were still reduced compared to control cells, although the difference was less pronounced (Figure 3, right panel).

No significant difference between the examined biological matrices was observed. Our data suggest that new collagen deposition seems unlikely during the analyzed timeframe.

#### 2.2.2. Expression and Activity of Extracellular Matrix Degrading Enzymes

Then we investigated if, in the same timeframe, specific matrix metalloproteinase levels were modified.

**Figure 4:**
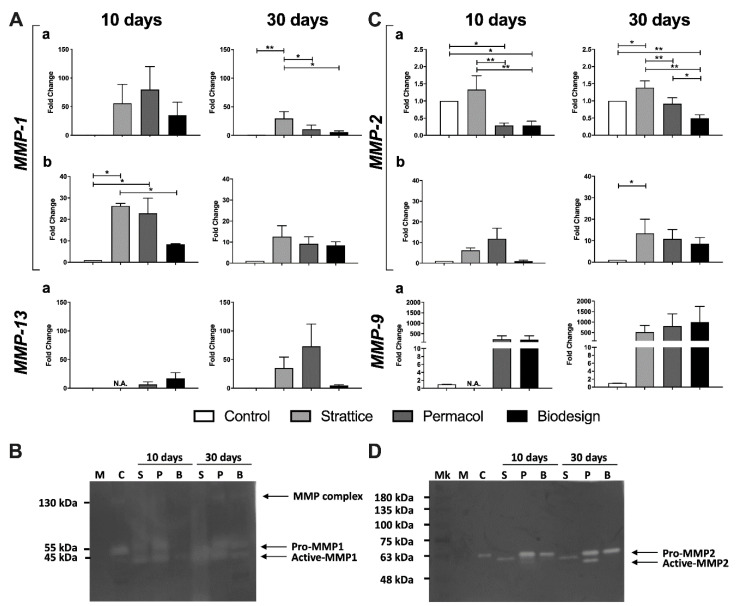
Changes in the levels of metalloproteinases transcripts, proteins, and in their activity following growth on the different types of matrices for 10 or 30 days, compared to control condition. (**A**) Expression of MMP-1 and MMP-13 collagenases from fibroblasts grown on plastics (control), Strattice, Permacol, Biodesign at transcript (a) and protein (b) levels. (**B**) Representative image of collagen zymography using supernatants from fibroblasts grown on plastics (C), Strattice (S), Permacol (P), and Biodesign (B) for 10 and 30 days. Complete medium (M) was used as a negative control. (**C**) Expression of MMP-2 and MMP-9 gelatinases from fibroblasts grown on plastics (control), Strattice, Permacol, Biodesign at transcript (a) and protein (b) levels. (**D**) Representative image of gelatin zymography using supernatants from fibroblasts grown on plastics (C), Strattice (S), Permacol (P), and Biodesign (B) for 10 and 30 days. Complete medium (M) was used as a negative control. Values in (**A**,**C**) are means of four independent experiments ± SE. The results were analyzed with ANOVA. Asterisks indicate significant differences (* *p* < 0.05; ** *p* < 0.01). N.A. indicates that the sample was not analyzed since the amount of RNA extracted from the sample was not sufficient to analyze all genes of interest.

We observed an increase in MMP-1 transcripts in fibroblasts grown on all 3D biological matrices compared to cells grown in 2D on plastics (Figure 4A). Due to the high variability in the biological replicates, after 10 days, the increase was not significant (Figure 4A); at 30 days, fibroblasts cultured on Strattice showed a significant increase in MMP-1, compared not only with control cells but also to cells grown on the other two biological matrices (Figure 4A, right panel). Moreover, at 30 days, MMP-1 expression by fibroblasts grown on Permacol and Biodesign scaffolds was also increased compared to that of control cells (Figure 4A). Measurement of MMP-1 protein in cell supernatants by enzyme-linked immunosorbent assay (ELISA) confirmed the results of MMP-1 transcript levels (Figure 4A). Fibroblasts grown on Strattice had the highest levels of this protease at both time points, whereas cells grown on Biodesign had the lowest increase among those grown on the three 3D matrices, but still higher than control cells (Figure 4A). We did not observe significant changes in MMP-13 transcripts (Figure 4A).

To investigate if, in the presence of 3D biological matrices, the proportion of active collagenases was also increased, we performed collagen zymography. Supernatants from fibroblasts grown in 2D on plastics expressed only pro-MMP1, whereas those from fibroblasts grown on biological matrices showed an equal proportion of pro- and active MMP-1, or even a preponderance of active form in the case of cells grown on Strattice at 30 days (Figure 4B). No evidence of MMP-13 activity was observed in these zymograms (Figure 4B). It must be said that the Cq values in qPCR were above 30 for this metalloproteinase even when induced, suggesting that levels of expression remained low (Supplementary Table S1). Our results suggest that MMP-13 expression and activity were negligible in the conditions tested.

MMP-2 transcript levels showed small but significant changes not only between controls and fibroblasts grown on biological meshes but also among the different matrices. Transcript levels were modestly but consistently increased in the Strattice samples compared to control at 30 days, and the increase was confirmed by quantification of protein levels (Figure 4C). On the contrary, cells grown on Biodesign showed a 50% decrease in the transcript when compared to control at both times. Interestingly, while no increase in MMP-2 protein was observed at 10 days compared to control cells, an eight-fold increase was measured in supernatants from cells cultured on the Biodesign sample for 30 days (Figure 4C). However, the difference described in MMP-2 protein levels was statistically significant only for Strattice. MMP-2 expression in cells grown on Permacol was more variable in time, and transcript and protein levels did not correlate (Figure 4C).

MMP-9 transcript levels were increased in cells grown on all biological matrices compared to control conditions, albeit not significantly, due to high variability among experiments. For cells grown on Strattice for 10 days, the number of cells did not allow to extract sufficient amounts of RNA to analyze all genes of interest (Figure 4C).

We then performed gelatin zymography to assay for the presence of pro- and active forms of MMP-2 and MMP-9 gelatinases. Supernatants from fibroblasts grown on plastics showed only pro-MMP-2, whereas fibroblasts grown on biological matrices showed either a shift towards the active form or an increase in the total activity of MMP-2, variably distributed between the two forms (Figure 4D). MMP-9 activity was not detected in our gels. Indeed, as for MMP-13, also for MMP-9, the levels of transcript were low, even after induction: the Cq from fibroblasts grown on plastics was above 35 and was no more than 30 when cells grown on biological matrices were analyzed (Supplementary Table S1). In conclusion, fibroblasts grown on biological matrices showed an increase in the expression and activity of only specific metalloproteinases (MMP-1 and MMP-2) during the first weeks of growth. For both enzymes, the expression was Strattice > Permacol > Biodesign.

#### 2.2.3. Expression of Metalloproteinase Inhibitors

**Figure 5:**
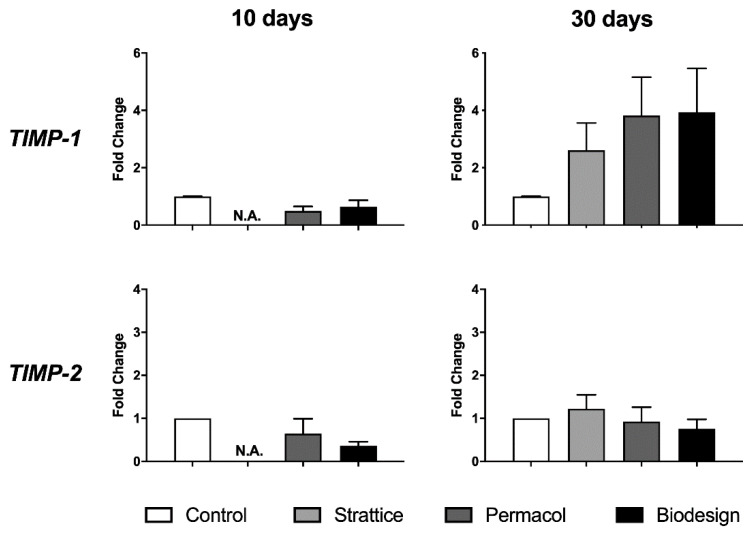
Changes in the transcript levels of metalloproteinase inhibitors following growth on the different types of matrices for 10 or 30 days compared to the control condition—expression from fibroblasts grown on plastics (control), Strattice, Permacol, and Biodesign. Values are the means of four independent experiments ± SE. The results were analyzed with ANOVA. N.A. indicates that the sample was not analyzed since the amount of RNA extracted from the sample was not sufficient to analyze all genes of interest.

Next, we investigated whether TIMP-1, MMP-1 specific inhibitor, was expressed by fibroblasts grown on meshes. Although a slight increase in TIMP-1 was observed at 30 days after cell seeding in all biological matrices compared to control cells, the difference was not significant (Figure 5). TIMP-2, analyzed as a control, did not show any change in the conditions tested (Figure 5).

#### 2.2.4. Expression of Cytokines and Alpha-Smooth Muscle Actin (α-SMA)

**Figure 6:**
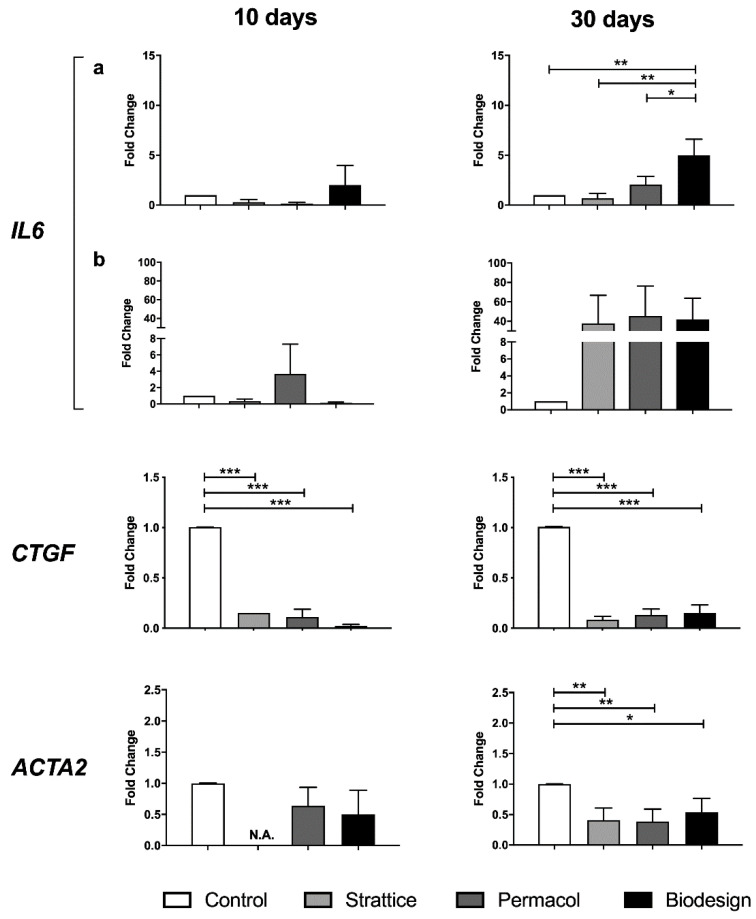
Changes in the levels of IL-6, CTGF, and α-smooth muscle actin following growth on the different types of matrices for 10 or 30 days, compared to the control condition. Expression from fibroblasts grown on plastics (control), Strattice, Permacol, Biodesign at transcript (**a**) and protein (**b**) levels. Values are the means of four independent experiments ± SE. The results were analyzed with ANOVA. Asterisks indicate significant differences (* *p* < 0.05; ** *p* < 0.01; *** *p* < 0.001). N.A. in transcript graphs (**a**) indicates that expression was not analyzed since the amount of RNA extracted from the sample was not sufficient to analyze all genes of interest.

Finally, we investigated if specific cytokines, namely Interleukin-6 (IL-6) and Connective Tissue Growth Factor (CTGF), were differentially expressed by fibroblasts grown on the different types of matrices. IL-6 transcript levels were increased, in particular in fibroblasts grown on the Biodesign samples at both 10 and 30 days. At 30 days, the difference was significant not only compared to control conditions but also compared to cells grown on the other types of matrices (Figure 6). Levels of interleukin-6 protein were not affected at 10 days by any of the matrices; however, an increase was observed at 30 days in supernatants from cells grown on all biological meshes compared to control (Figure 6, right panel).

CTGF is considered a marker of myofibroblast differentiation together with alpha-smooth muscle actin (α-SMA), the product of the *ACTA2* gene. Expression of both genes was examined to investigate the possible differentiation of fibroblasts, cultured on the different scaffolds, into myofibroblasts, responsible for the induction of the fibrotic reaction. A highly significant and consistent reduction in CTGF transcript was observed in fibroblasts grown on all types of biological matrices compared to cells grown on plastics for the whole duration of the experiment (Figure 6).

Also, *ACTA2* transcript levels were reduced in fibroblasts grown on biological matrices compared to cells grown on plastics, in particular after 30 days of growth (Figure 6).

Cumulatively, these results suggest that fibroblasts do not undergo differentiation into myofibroblasts when grown on 3D biological matrices in the studied timeframe.

## 3. Discussion

Surgical repair of the abdominal wall with the insertion of prosthetic materials has become a common procedure for the treatment of hernias or lesions of the muscular inner body walls. Recently, the same matrices have also been applied in breast reconstructive surgery [2]. Several materials are available to date, making the choice difficult. As several factors intervene to influence the outcome (the type of surgery needed, original lesion, the lifestyle of the patient, and comorbidities), it is very difficult to summarize indications even with extensive metanalysis studies [4,13,14].

Biological matrices are derived from the extracellular matrix of a variety of tissues and species and undergo extensive processing to make them suitable for implantation; this processing can affect matrix properties and induce foreign body response (FBR) [6,15,16]. It has been proposed that biological matrices are more resistant than synthetic meshes to microbial infection, have the ability to provide a barrier to visceral adhesion formation, and to respond in a fashion similar to native tissue [7,8]. However, these desired features have not been investigated in detail.

In this work, we aimed to investigate the behavior of human fibroblasts, the most abundant cell type in connective tissues, when cultured in vitro in the presence of biological 3D matrices. Several aspects of the interaction of human primary fibroblasts from derma of healthy adult individuals with biological scaffolds in vitro were studied by means of cell counting, SEM, and gene expression analyses. We tested three types of biological mesh commonly used for hernia repair that differed in the tissue of origin and/or presence of crosslinking, together with a synthetic mesh, used for comparison. The physicochemical properties of the materials have been previously described by Deeken et al. [17]. Insertion of prosthetic materials can occur at different anatomical positions depending on the type of surgery. Thus, different fibroblast subtypes may be involved in the interaction. However, given the scarce knowledge about the different behavior of these cell subtypes, mainly obtained in model animals, the availability of cells, and the need to obtain several replicates, we focused our work on human dermal fibroblasts [18,19].

In our study, fibroblasts showed different ability to attach and grow on the different materials, with the following order: Biodesign > Permacol > Strattice > Prolene. Although on the Biodesign samples, the number of cells was initially higher, at 30 days, it was comparable to that of the other matrices. This number may represent the maximum number of cells that could grow on these matrices; however, we did not observe confluence on the other types of matrices. An alternative explanation is that on the Biodesign matrix, cells loosely interacting with the surface were lost during the processing of Biodesign samples. Indeed, at increasing cell density, fibroblasts formed a cell monolayer, and the interaction with this matrix appeared weakened (Figure 1l). Cells firmly adhered and proliferated on Strattice and Permacol but showed a non-homogeneous distribution. Our morphological analysis under the scanning electron microscope seems to confirm that the strongly oriented collagen of the submucosal scaffold (Biodesign) may be the reason for a greater elongation and alignment of the fibroblasts if compared to the multi-directionally oriented collagen on dermal Permacol and Strattice scaffolds. The fact that collagen fibers’ direction may influence cell growth has already been reported [20,21,22,23]. In addition, the differences in cell numbers that we observed between the two meshes derived from porcine derma may be due to the different procedures used for the preparation of matrices before commercialization.

The observation that human dermal fibroblasts bound less efficiently than rat kidney fibroblasts or mesenchymal stem cells (MSC) to Strattice has been reported previously [19]. In the same work, fibroblasts failed to coat Marlex, a small pore, monofilament, polypropylene mesh, confirming the results we obtained with Prolene, a similar material [19]. Also, in vivo in animal models, Strattice was described to undergo poor incorporation; however, no adhesion formation was observed [5].

Our study highlighted similarities and differences in the gene expression programs activated by fibroblasts grown on the different matrices. The first similarity was observed in the reduction of collagen transcripts in cells grown on all biological matrices compared to control cells in the investigated timeframe. However, the observation that at 30 days, the reduction in expression was less marked than at 10 days suggests that expression may increase later in time. Interestingly, collagen deposition seems to be a late event, also in an in vivo mouse model [24].

Fibroblasts grown on biological matrices showed a decreased expression of markers of fibrosis, such as Connective Tissue Growth Factor (CTGF), alpha-SMA, and collagen I, compared to control cells [3]. α-SMA—product of the *ACTA2* gene—is known to increase during tissue remodeling and fibrosis, when fibroblasts differentiate into myofibroblast, and its expression is associated with up-regulation of collagen I and fibronectin biosynthesis and down-regulation of matrix remodeling enzymes [25,26,27,28]. CTGF is involved in adhesion, migration, and proliferation of fibroblasts, but also in myofibroblast differentiation and fibrotic disease [29,30]. Since myofibroblast differentiation occurs during the fibrotic processes and is strictly related to the amount of foreign body reaction (FBR) induced at the biomaterial/host-tissue interface, the lack of expression of these markers in cells grown on 3D matrices compared to control cells suggests that this type of differentiation does not occur in the timeframe we analyzed. It would have been interesting to compare the expression of the same genes in cells grown on the synthetic mesh; unfortunately, we did not retrieve sufficient cells for molecular analyses from this mesh. Prolene is a small pore polypropylene scaffold and, although this material is commonly used in surgery, it is increasingly recognized that it can increase recurrence rates and comorbidities, including fibrosis, compared to large pore meshes [31].

Our study also highlighted differences among matrices in the expression of specific genes. We found increased expression of MMP-1 in fibroblasts grown on all biological matrices both at the transcript and protein levels. Moreover, zymography showed that supernatants from fibroblasts grown on meshes are enriched in the active form of this collagenase. Also, MMP-2 was slightly increased at the protein level, as evident by ELISA and zymography. Although we found an increase in MMP-9 and MMP-13 at the transcript level, we did not find evidence of their activity in zymograms. Indeed, this is in keeping with the low starting level of these metalloproteinases when one considers the raw qPCR data in terms of cycle threshold (Supplementary Table S1). Published data confirm our findings, reporting that fibroblasts grown on 3D collagen express MMP-1 and MMP-2, but not MMP-9 [26]. High levels of MMP-2 expression and activity are known to promote fibroblast migration and increase angiogenesis during the proliferative phase of wound healing [32]. Since the expression of TIMP-1, targeting MMP-1, increased only slightly at 30 days in fibroblasts grown on all investigated 3D biological scaffolds, we hypothesized that matrix degradation exceeded collagen biosynthesis and degradation was not (yet) inhibited by TIMP production in the considered time frame. The activity of metalloproteinases could be involved in the degradation of the old collagen of the 3D matrices to be replaced with the newly synthesized matrix and to favor the colonization of fibroblasts.

Among the various cytokines regulating the expression of MMPs and TIMPs during wound healing [33,34], IL-6 is involved in fibroblast proliferation and fibroproliferative disorders [35]. Although cytokines are primarily produced by monocytes/macrophages at the wound site [36,37], there is evidence of an autocrine production by fibroblasts [38,39]. We found an increase in IL-6 protein in fibroblasts grown in the presence of all biological matrices, although the increase in the relative transcript was observed only in the Biodesign 3D scaffold after 30 days of culture.

Our data show that fibroblasts can grow on biological matrices used for surgical applications and adapt their transcriptional program to the presence of these materials. We found that they induce first matrix degrading enzymes, independently of the tissue of origin of the biomaterials or the presence of crosslinking; however, cells grown on Strattice expressed the highest levels of these enzymes. On the contrary, cells grown on Biodesign showed the highest levels of IL-6. Although it has been suggested that crosslinking may interfere with mesh incorporation in vivo [40,41,42], we did not find evidence of different cell growth or gene expression of the crosslinked matrix (Permacol) compared to the other materials in vitro. In order to mimic an in vivo interaction of fibroblasts with other cell types, such as macrophages, coculture experiments should also be performed.

The main limitation of this work is that it was an in vitro study. In vivo, the outcome of the insertion of prosthetic material is likely the result of the interaction of several cell types, including fibroblasts and monocytes/macrophages, which stimulate each other to produce and secrete cytokines and are capable to attract endothelial cells, stimulating angiogenesis and incorporation of meshes into host tissue [36,37]. Integration of data from in vitro and in vivo work (both in animal models and from human explants) will allow further understanding of the interaction between host cells and different types of matrix, to be able to choose the right material for each patient in clinical applications.

## 4. Materials and Methods

### 4.1. Cell Culture

Human dermal fibroblasts from healthy adult donors (aged 35–45) were obtained from the “Cell Line and DNA Biobank from Patients Affected by Genetic Diseases—NETWORK OF GENETIC BIOBANKS TELETHON” [43]. Fibroblasts were used for the experiments between passages 11 and 13. The experiments were performed using fibroblasts from two different donors.

Fibroblasts were grown in RPMI-1640 (Carlo Erba Reagents, Milan, Italy) supplemented with 10% Fetal Bovine Serum (FBS, Carlo Erba Reagents, Milan, Italy) and 2 mM L-Glutamine (Carlo Erba Reagents, Milan, Italy). Cultures were incubated at 37 °C in 95% humidity and 5% CO_2_ atmosphere. At confluence, fibroblasts were detached from culture flasks by treatment with trypsin (Carlo Erba Reagents, Milan, Italy). Cells were thawed on an average of two weeks before each experiment to have sufficient numbers of cells. Cells were routinely checked for the presence of mycoplasma by using the nested PCR method described in Tang et al. [44].

### 4.2. Type and Preparation of Matrices

The matrices used in this study are described in Table 1 and were obtained from the manufacturing companies. The Prolene synthetic mesh was included for comparison.

The matrices were cut into small pieces (1 cm × 1 cm): Strattice and Permacol were washed with PBS (Phosphate Buffered Saline, Carlo Erba Reagents, Milan, Italy) for eight hours at room temperature with gentle shaking, changing the washing every 2 h, whereas Biodesign and Prolene were washed at room temperature for 25 min with gentle shaking, changing the washing every 5 min. The matrices were placed in 12 well tissue culture plates containing complete medium and were incubated for 12 h at 37 °C in 95% humidity and 5% CO_2_ atmosphere.

### 4.3. Experimental Setup

Twelve hours after preparation of the matrices, 5 × 10^4^ fibroblasts were seeded on matrices’ surface in each well and then incubated for two days at 37 °C in 95% humidity and 5% in a CO_2_ atmosphere. Then, the matrices were transferred to a new tissue culture plate, and fibroblasts were allowed to grow for 10, 20, and 30 days.

Three days before the end of the treatment, the serum-containing culture medium was replaced with RPMI-1640 supplemented with 2 mM L-Glutamine and Lyset Kit 5% (Sclavo Diagnostic International by Carlo Erba Reagents, Milan, Italy) using a ratio of 1:4 among the Lyset PL: AD reagents (PL: human platelet lysate from platelet-rich plasma; AD: human platelet-poor plasma), to have more controlled growth factor and cytokine composition.

For metalloproteinase zymography, the growth medium was replaced with serum-free medium (RPMI-1640 supplemented with 2 mM L-glutamine) two days before the end of treatment. For zymography experiments, the serum-free conditioned medium was collected.

The supernatants for ELISA and metalloproteinase zymography were collected at the end of the treatments and stored at −80 °C. The matrices were used for RNA extraction, scanning electron microscopy, and cell counting.

The control condition was represented by fibroblasts grown in “2D” on plastics, with the same media described above. Since the cells on plastics proliferate faster and have to be passed while the cells on the biomaterials grow more slowly, we processed control cells when 80% confluent after the start of each experiment.

Three independent experiments were performed for scanning electron microscopy analysis and for cell counting; four independent experiments were performed for gene expression analyses/zymography.

### 4.4. Scanning Electron Microscopy (SEM)

Samples were washed in PBS and fixed for 1 h in Karnovsky fixative (2% paraformaldehyde and 2.5% glutaraldehyde in 0.1 M cacodylate buffer (pH 7.2)), washed in 0.1 M cacodylate buffer (pH 7.2) and subsequently postfixed for 2 h in 1% osmium tetroxide and potassium ferrocyanide in 0.1 M cacodylate buffer (pH 7.2). After a series of washes with PBS (pH 7.2), the samples were immersed for 1 h in osmium tetroxide (0.1% in PBS). After being dehydrated in an increasing series of ethanol (70%, 90%, and 100%), the samples were dried with hexamethyldisilazane at the critical point. Once mounted on stubs, the samples were coated in gold with a Sputter K250 (Emitech, Baltimore, MD, USA) and subsequently observed with a SEM-FEG XL-30 microscope (Philips, Eindhoven, The Netherlands).

### 4.5. Cell Counting

The number of cells grown on the different scaffolds was determined by two methods: the first, CellTiter-Glo kit (Promega, Milan, Italy), is based on ATP quantification and was performed following manufacturer instructions after direct lysis of cells on matrices. A calibration curve was prepared with known amounts of cells to estimate the number of cells retrieved from each matrix; the second method consisted of enzymatic detachment of cells from the matrices, followed by manual cell counting. Cell containing matrices were incubated for 25 min at 37 °C with an enzymatic cocktail, composed of Collagenase from Clostridium Histolyticum, Type IA (Sigma-Aldrich, Milan, Italy), at a final concentration of 5 mg/mL, directly diluted in Accutase solution (Carlo Erba Reagents, Milan, Italy). Then 100 µL of trypsin was added for 5 min at 37 °C. Cells were counted with a hemocytometer after adding a volume of trypan blue.

### 4.6. RNA Extraction and qPCR Analysis

3D matrices were manually scraped in 300 µL of TriReagent (Sigma-Aldrich, Milan, Italy) on ice to retrieve fibroblasts; then lysates were collected in tubes and stored at −80 °C. Fibroblasts grown on tissue culture plates were used as the control condition. Total RNA was extracted using a Direct-zol RNA MiniPrep (Zymo Research, EuroClone, Milan, Italy) following manufacturer instructions. RNA samples were quantified with a NanoDrop 2000c (ThermoFisher, Life Technologies Italia, Milan, Italy) and run on an agarose gel. For real-time quantitative PCR (qPCR), cDNA was obtained from 750 ng of RNA by using the iScript cDNA synthesis kit (Biorad, Milan, Italy). Gene expression analyses were performed in triplicate using a CFX96 thermal cycler (Biorad, Milan, Italy) and the iTAQ Universal Sybr Green Supermix (Biorad, Milan, Italy). Relative mRNA quantification was obtained by applying the 2^−∆∆Cq^ method [45], using the geometric average of the Cqs of two reference genes, namely beta-2-Microglobulin and GAPDH, for normalization purposes. Melting curve analysis was performed to ensure that single amplicons were obtained for each target. Primers for the genes under investigation were designed to have at least one of the primers in the pair designed on an exon-exon junction or to encompass at least one intron. Primer sequences are reported in Supplementary Table S2.

### 4.7. Quantification of Secreted Proteins by ELISA

The concentrations of human MMP1, MMP2, and IL-6 in cell culture supernatants from fibroblasts grown on the different types of matrices were measured using Human MMP1 (ThermoFisher Scientific, Milan, Italy), Human MMP2 (Novex, Life Technologies Italia, Milan, Italy) and Human IL-6 (ThermoFisher Scientific, Milan, Italy) colorimetric ELISA kits, following manufacturer’s instructions. The obtained ELISA measurements were normalized by cell number. The two ELISA kits for MMP-1 and MMP-2 recognize both the pro- and the active forms of each metalloproteinase.

### 4.8. Zymography Analysis

To evaluate the enzymatic activity of metalloproteinases, zymography was performed. Supernatants from cells cultured as described before preparation for electrophoresis by adding one volume of non-reducing sample buffer (250 mM Tris HCl pH 6.8, 40% glycerol, 8% SDS, 0.01% bromophenol blue) every three volumes of supernatant. The appropriate volumes of supernatant were calculated after normalization by cell number. Samples were loaded on eight percent polyacrylamide gels containing 5 mg/mL of gelatin from bovine skin (Sigma, Milan Italy) or 1 mg/mL of collagen type I from rat tail (Sigma, Milan, Italy). After electrophoresis, gels were washed for 1 h in 2.5% Triton X-100 at room temperature and then incubated at 37 °C in 10 mM CaCl_2_, 150 mM NaCl and 50 mM Tris HCl pH 7.5 overnight for gelatinases and for 48 h for collagenases. At the end of incubation, gels were stained with 0.25% Coomassie brilliant blue G-250 in 50% methanol, 10% acetic acid, and then destained with a solution of 10% isopropanol and 10% acid acetic until metalloproteinase activity was detected as white bands in the gel.

### 4.9. Statistical Analysis

The numbers of cells grown on the different matrices were compared using analysis of variance (ANOVA) with *post-hoc* Bonferroni test (GraphPad Prism statistical software program, version 4.02, San Diego, CA, USA). Data from gene expression analysis and from quantification of secreted proteins from four independent experiments were compared using analysis of variance (ANOVA) with *post-hoc* Duncan’s test (Statistica data analysis and visualization program, version 8.0, Hamburg, Germany). The differences in cell numbers grown on the different matrices at different times and the differences in protein secretion evaluated by ELISA were compared using analysis of variance (ANOVA) (GraphPad Prism statistical software program, version 4.02, San Diego, CA, USA).

## 5. Conclusions

The purpose of this study was to compare the growth of human primary dermal fibroblasts on 3D matrices commonly used for surgery and the activation of specific molecular pathways that may indicate the first processes occurring in the early stages of matrix implantation.

Our gene expression results suggest that biological matrices may be absorbed and substituted by endogenously produced extracellular matrix and that, in spite of an overall similar transcriptional program, there are some differences among the tested matrices that may influence the rate of implant engraftment and could even be exploited in particular instances.

## Figures and Tables

**Table 1:** Description of matrices used in this study.

Trade Name	Manufacturer	Species	Tissue Type	Crosslinked	Sterilization
Strattice	LifeCell-Acelity	Porcine	Dermis	No	E-beam
Permacol	Covidien	Porcine	Dermis	Yes (hexamethylene diisocyanate)	Gamma irradiation
Surgisis-Biodesign	Cook-Medical	Porcine	Small intestine submucosa	No	Ethylene oxide
Prolene Polypropylene Mesh	Ethicon	Synthetic	Polypropylene high weight, monofilament	N.A. ^(a)^	Ethylene oxide

^(a)^ N.A. Not applicable.

## Data Availability

The data presented in this study are available within the article and its supplementary material.

## Supplementary Materials

The following are available online at https://www.mdpi.com/1422-0067/22/2/526/s1. Figure S1. Morphological analysis of unseeded matrices and of fibroblasts grown on tested matrices for 10 or 30 days; Table S1. Average Cq and standard error for each metalloproteinase under investigation and for the reference genes used in this work; Table S2. Sequences of primers used for gene expression analysis in this study.

## Abbreviations

2D	Two dimensional
3D	Three dimensional
α-SMA	Actin, alpha 2, smooth muscle protein
*ACTA2*	Actin, alpha 2, smooth muscle gene
ANOVA	Analysis of variance
*B2M*	Beta-2-microglobulin
*COL1A1*	CollCollagen type I alpha 1 chain
*COL1A2*	CollCollagen type I alpha 2 chain
*COL3A1*	Collagen type III alpha 1 chain
Cq	Cycle quantitative (PCR cycle number at which the amplification curve intersects the threshold line)
*CTGF*	Connective tissue growth factor
ECM	Extracellular matrix
ELISA	Enzyme-linked immunosorbent assay
FBR	Foreign body response
*GAPDH*	Glyceraldehyde-3-phosphate dehydrogenase
*IL-6*	Interleukin 6
MMP	Metalloproteinase
MSC	Mesenchymal stem cell
SEM	Scanning Electron Microscopy
TIMP	Metallopeptidase inhibitor
