# Supplementary material for: Human Primary Dermal Fibroblasts Interacting with 3-Dimensional Matrices for Surgical Application Show Specific Growth and Gene Expression Programs
Source: Int J Mol Sci. 2021 Jan 7;22(2):526. doi: 10.3390/ijms22020526 (PMC7825678; doi:10.3390/ijms22020526)
Supplement: Supplementary file 1 [file ijms-22-00526-s001.pdf]

Figure S1. Morphological analysis of unseeded matrices and of fibroblasts grown on tested matrices for 10 or 30 days. Scanning Electron Microscopy (SEM) images at high magnification are shown. a-c Strattice; d-f Permacol; g-i Biodesign; l-n Prolene. Bars: 100 μm.

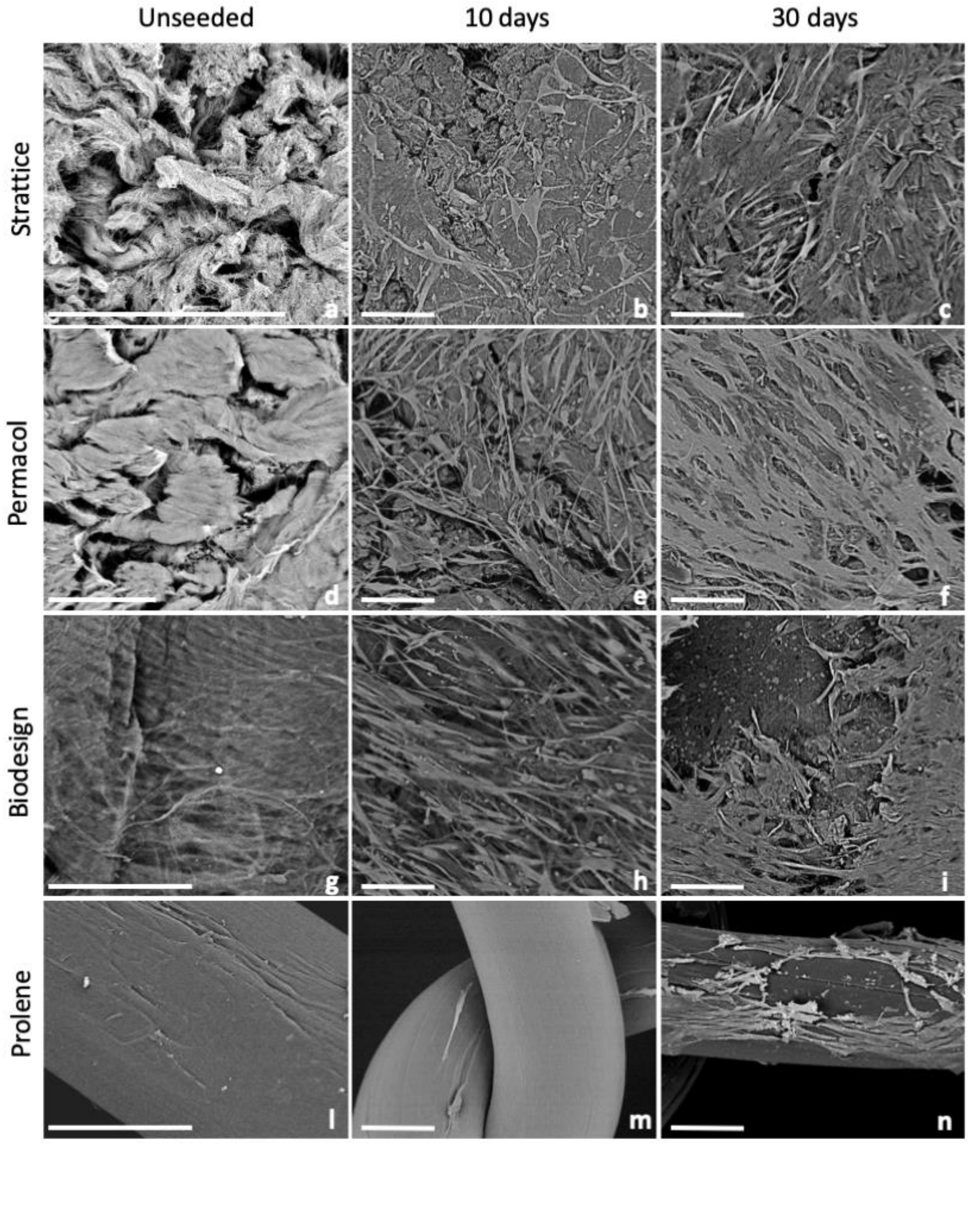

Table S1. Average Cq and standard error for each metalloproteinase under investigation and for the reference genes used in this work

The indicated values are the average of all technical (3 for each experiment) and biological replicates (*n*=4) performed for qPCR analyses for each condition in which fibroblasts were grown ± Standard Error. Control is represented by fibroblasts grown on plastics as described in Materials and Methods.

	MMP1		***MMP2***		***MMP9***		***MMP13***		***B2M+GAPDH***	
	**10 days**	**30 days**	**10 days**	**30 days**	**10 days**	**30 days**	**10 days**	**30 days**	**10 days**	**30 days**
**Control**	22,81±0,60		20,29±0,17		37,72±0,63		34,72±0,72		17,79±0,34	
**Strattice**	16,69±0,05	19,33±0,49	18,49±0,12	20,71±0,19	N.A.	31,17±0,82	N.A.	31,59±0,24	16,16±0,01	18,66±0,34
**Permacol**	18,10±0,28	21,19±0,38	22,88±0,14	21,31±0,15	31,73±0,67	30,55±0,48	33,60±0,09	30,63±0,31	18,48±0,16	18,65±0,34
**Biodesign**	20,92±1,13	22,19±0,28	24,83±1,15	22,92±0,24	36,47±1,77	30,96±0,64	36,03±0,48	33,98±0,80	20,00±0,85	19,47±0,45

Table S2. Sequences of primers used for gene expression analysis in this study.

Gene	Ref_Seq		Sequence (5' → 3')	Product length (bp)
MMP1	NM_001145938.1; NM_002421.3	F	AAGGTCTCTGAGGGTCAAGCA	59
		R	TCCCGATGATCTCCCCTGAC	
MMP2	NM_001127891.2; NM_001302508.1; NM_001302509.1; NM_001302510.1; NM_004530.5	F	GCCAAGTGGTCCGTGTGAA	86
		R	GCTGTTGTACTCCTTGCCATTG	
MMP9	NM_004994.2	F	TTCTGCCCGGACCAAGGATA	89
		R	TCCGGCACTGAGGAATGATCT	
TIMP1	NM_003254.2	F	GCAATTCCGACCTCGTCATCA	134
		R	GTCAGCGGCATCCCCTAAG	
TIMP2	NM_003255.4	F	GCTGCGAGTGCAAGATCAC	108
		R	GGTGCCCGTTGATGTTCTTC	
MMP13	NM_002427.3	F	GGAATTAAGGAGCATGGCGAC	76
		R	GCCCAGGAGGAAAAGCATGA	
COL1A1	NM_000088.3	F	CAAGACGAAGACATCCCACCAA	128
		R	ACGTCATCGCACAACACCTT	
COL1A2	NM_000089.3	F	TGAAGATGGTCACCCTGGAAAA	65
		R	CACCCTGTGGTCCAACAACT	
COL3A1	NM_000090.3	F	TCGAGGCAGTGATGGTCAAC	90
		R	GGTCCAACTTCACCCTTAGCA	
CTGF	NM_001901.2	F	TGCACCGCCAAAGATGGT	148
		R	GCAGACGAACGTCCATGCT	
IL6	NM_000600.4; NM_001318095.1	F	TAGTGAGGAACAAGCCAGAGC	104
		R	TTGGGTCAGGGGTGGTTATTG	
ACTA2	NM_001141945.1; NM_001613.2	F	GGCAAGTGATCACCATCGGA	100
		R	GTGGTTTCATGGATGCCAGC	
GAPDH	NM_001289746.1; NM_001289745.1; NM_002046.5	F	GAAGGTGAAGGTCGGAGTC	226
		R	GAAGATGGTGATGGGATTTC	
B2M	NM_004048.2	F	AGGCTATCCAGCGTACTCCA	102
		R	ATGGATGAAACCCAGACACA	
